# Optimal Tuning of Random Survival Forest Hyperparameter with an Application to Liver Disease

**DOI:** 10.21315/mjms2022.29.6.7

**Published:** 2022-12-22

**Authors:** Kazeem Adesina Dauda

**Affiliations:** Department of Statistics and Mathematical Sciences, Kwara State University, Malete, Nigeria

**Keywords:** survival time, censoring time, classical model, primary biliary cirrhosis, Random Survival Forest

## Abstract

**Background:**

Random Forest (RF) is a technique that optimises predictive accuracy by fitting an ensemble of trees to stabilise model estimates. The RF techniques were adapted into survival analysis to model the survival of patients with liver disease in order to identify biomarkers that are highly influential in patient prognostics.

**Methods:**

The methodology of this study begins by applying the classical Cox proportional hazard (Cox-PH) model and three parametric survival models (exponential, Weibull and lognormal) to the published dataset. The study further applied the supervised learning methods of Tuning Random Survival Forest (TRSF) parameters and the conditional inference Forest (Cforest) to optimally predict patient survival probabilities.

**Results:**

The efficiency of these models was compared using the Akaike information criteria (AIC) and integrated Brier score (IBS). The results revealed that the Cox-PH model (AIC = 185.7233) outperforms the three classical models. We further analysed these data to observe the functional relationships that exist between the patient survival function and the covariates using TRSF.

**Conclusion:**

The IBS result of the TRFS demonstrated satisfactory performance over other methods. Ultimately, it was observed from the TRSF results that some of the covariates contributed positively and negatively to patient survival prognostics.

## Introduction

Survival analysis is quite common in clinical study and some other related fields. It is used to study the occurrence of some events of subjects and time until the events occur. The time is known as the survival time or failure time at which the event of interest occurs. It can be measured in days, months and/or years depending on the type of experiment, while the event can be death, alive, replace and so on from any kind of disease, such as liver disease (1).

There are various established classical modeling approaches to survival analysis as found in the literature. Parametric models are one of these and are based on specified families of the distributions that involve stringent and strict assumptions on the survival time, which usually simplify the experimental evidence in the clinical trial experiment (2–4). The second classical model, which is the most commonly used model among survival analysis techniques is a semiparametric model popularly known as the Cox proportional hazard (Cox-PH) model, which as developed by David Cox (5). The Cox-PH model does not make assumptions about the distribution of failure time but instead makes assumptions on how covariates influence the survival time. This assumption is regarded as a ‘proportionality hazard’ assumption, that is, the effect of every covariate is constant over time (5, 6). Additionally, the Cox-PH model does not allow the direct estimation of survival times (2). Finally, these parametric and semiparametric methods were developed to investigate the possible relationship between the survival time and the various covariates. However, when the underlying assumptions that govern these techniques are not satisfied, the models may not yield reliable and faithful conclusion. Therefore, non-parametric models such as survival tree (7, 8), Random Forest (RF) (9–13), neural network (14, 15) and multivariate adaptive regression splines (16, 17) have evolved to circumvent the restrictive assumption problems.

Recently, ensemble-based approaches that combine both parametric and non-parametric models with ensemble learning techniques have been applied to censored data to create accurate and diverse base learners. Some of these ensemble-based approaches that are applied to survival trees include bagging (18, 19), boosting (20), RF (21–23) and the conditional inference Forest (Cforest) (24).

In this article, we focus on modifying Random Survival Forests (RSFs) by introducing the tuning parameter to the hyperplane of the trees to increase the predictive accuracy of the RSF. The proposed Tuning Random Survival Forest (TRSF) methodology extends the original RSF algorithm to censored data and implements the grid search method to obtain optimal parameters.

The parameters of interest to be tuned are the number of variables at each split and the minimum number of unique observations needed to split a node, which will be done by grid search methods. More precisely, we will investigate the efficiency of TRSF on real-life data and compare our results with the classical methods.

## Methods

Let the observations of each subject *i* denote (*T_i_*, *δ_i_*, *x_i_*) where *T_i_* is the observed survival time *t* for subject *i*, *δ_i_* is the censoring index with value 0 if right-censored and 1 if experienced the event of interest, and *x_i_* is the vector of covariates, assuming *U* and C are the true time-to-event and the true censoring time for subject *i*. Hence, the observed time *T_i_ =* min (U, C) and *δ_i_ = I*(*U* ≤ *C*) . Usually, *U* and *C* are assumed to be independent, that it is the true time-to-event and the true censoring time is independent given the covariates.

### Cox-PH Model

The common model that predicts the hazard function of subject *i* is the Cox-PH model, which specifies the conditional cumulative hazard function depending on the vector of covariates:


(Equation 1) $$\wedge (t|X)=\wedge_{0}(t)\;exp\;(\beta^{T}X)$$

where ∧_0_ (*t*) is the cumulative baseline hazard and *β* = *β*_1_, *β*_2_, ... , *β*_K_) *∈* ℝ*^K^* is the unknown vector regression coefficients. The coefficients in Equation 1 can be estimated by maximising the partial likelihood with a modified risk set and inverse probability of censoring weights (5). Thus, the partial likelihood and the score function of the model (1) are given as:


(Equation 2) LP(β)=∏ti=1n[βeTXi∑jɛRiβTXj]δi

where *R_i_* is the risk set at the time *t_i_*. The log-partial likelihood is:


(Equation 3) $$l_{P}(\beta )=\sum_{t_{i}=1}^{n}\delta_{i}\left[\beta^{T}X_{i}-\text{log}\left\{\sum_{jɛR_{i}}\beta^{T}X_{j}\right\}\right]$$

Then, the partial likelihood score function is:


(Equation 4) $$U(\beta )=\frac{d_{l}P(\beta )}{d\beta }=\sum_{t_{i}=1}^{n}\delta_{i}\left[X_{j}-\text{log }\left\{\frac{\sum_{jɛR_{i}}X_{j}e^{\beta^{T}X_{j}}}{\sum_{jɛR_{i}}\beta^{T}X_{j}}\right\}\right]$$

Thus, the maximum partial likelihood estimator can be found by solving *U* (*β*) = 0.

In what follows, we consider the implementation of an RSF algorithm (22, 25).

### Structure of Random Survival Forest

The RSF was designed to build many binary trees; however, the major aggregation scheme is based on a cumulative hazard function (CHF) described in Equation 1. The steps of this algorithm are as follows:

- Draw bootstrap samples from the original data *ntree* times. For each bootstrap sample, this leaves approximately one-third of the samples out-of-bags (OOB).
- A survival tree is grown for each bootstrap sample.
  - At each node of the tree, randomly select the square root of number of independent variables for splitting.
  - Using the log-rank-based splitting criteria described below, a node is split using the single covariate from step ii (a) that maximises the survival differences between daughter nodes.
  - Repeat steps ii (a) and ii (b) until each terminal node contains no more than 0.632 times the number of events.
- Calculate a CHF for each survival tree built. Aggregate the *ntree* trees to obtain the ensemble’s cumulative hazard estimate.

### Grid Search Method

The grid search method is an alternative method used for finding the best parameter for the model so that the classifier can accurately predict the unlabeled data. This method is categorised as an exhaustive method for the best parameter values that must each be explored, each by setting sort of prediction values at first. Then, the method will show the score value for each parameter value to consider which one will be chosen. These techniques will be applied to the algorithm above at step ii (a) for proper identification of the best parameter.

### Splitting Criteria

There are different splitting rules available in the literature, but we focus on using the log-rank-based criteria. The log-rank test for splitting is defined as follows:


(Equation 5) $$LR(X,c)=\frac{\sum_{i=1}^{N}d_{t^{i},{child}_{i}}-R_{t_{i},{child}_{1}}\frac{d_{t_{i}}}{R_{t_{i}}}}{\sqrt{\sum_{i=1}^{E}\frac{d_{t_{i}}\left(R_{t_{i}}-d_{t_{i}}\right)}{R_{t_{i}}-1}\frac{R_{t_{i},{child}_{1}}}{R_{t_{i}}}\left(1-\frac{R_{t_{i},{child}_{1}}}{R_{t_{i}}}\right)}}$$

where *N* is the number of distinct event times *T*_(1)_ ≤ *T*_(2)_ ≤ ... ≤ *T*_(_*_N_*_)_ in the parent node, is the number of an event at risk and at *d_ti,child j_* time *t*_1_ in the child nodes, *j* = 1, 2, *R_ti,child j_* is the number of individuals at risk at the time *t*_1_ in the child nodes, and *j* = 1, 2 is, the number of individuals who are alive or dead at the time *t*_1_, and *R_ti_* = *R_ti,child_*__1__ + *R_ti,child_*__2__ and *d_ti_* = *d_ti,child_*__1__ + *d_ti,child_*__2_._ It should be noted that the absolute value of *LR*(*X*,*c*) measures the node separation and the best split is chosen in such a way that it maximises the absolute value of:


(Equation 6) $$LRS(X,c)=\frac{\sum_{x_{i}\leq c^{a^{i}}}-n\mu_{a}}{\sqrt{n_{1}\left(1-\frac{n_{1}}{n}\right)}S_{a}^{2}}$$

where *μ_a_* and $s_{a}^{2}$ are the sample mean and sample variance of *a_i_*, respectively. Therefore, the main function of Equation 6 is to measure node separation based on cut-point *c*.

### Ensembles of Cumulative Hazard Function

When the survival reaches step (iii) in the algorithm, the trees are aggregated to form an ensemble CHF which is calculated by grouping the hazard estimate using terminal nodes. Suppose that *L* is the terminal node, *t_i,L_* is the distinct survival times, *d_ti,L_* is the number of events and *R_ti,L_* is the individual at risk at the time (*t_i,L_*). Thus, the CHF estimate for terminal node *L* is the Nelson-Aalen (26) estimator given by:


(Equation 7) $$\hat{\wedge }L\left(t\right)=\sum_{t_{i},L\leq t}\frac{d_{t_{i},L}}{R_{t_{i},L}}.$$

All individuals within *L* will have the same CHF. For *q* terminal nodes in a tree, there are *q* different CHF values. To determine ∧̂*L*(*t*) for an individual *i* with covariate *x_new_*, drop the tree and the *x_new_* will fall into a unique terminal node, L *∈* Q CHF at *L* would be the CHF for individual *i* in the test sample. The bootstrap for individual *i* is:


(Equation 8) $$\wedge^{*}\left(t|x_{new}\right)=\frac{1}{ntree}\sum_{b=1}^{ntree}\wedge_{b}^{*}(t|xnew)$$

where $\wedge_{b}^{*}(t|xnew)$ is the CHF for a particular tree. For the covariate, ensemble survival is defined as:


(Equation 9) $$S\left(t|x_{new}\right)=e^{-\wedge^{*}\left(t|x_{new}\right)}.$$

### Model Performance Indices

The performance of the proposed TRSF is designed to rely solely on nested (double) cross-validation (CV). The algorithm of nested cross-validation (Nested CV) (27) is divided into two categories (inner and outer loops); the first category (inner loops) is used in the study to prevent the hyperparameter from overfitting the data and this is called tuning of hyperparameter (Gridsearch CV). The second category (outer loop) is referred to as prediction accuracy or error rate (model performance); this study adopted the use of the integrated Brier score (IBS) (28) as a predictive accuracy measure. Within this section, the two categories of the Nested CV algorithm are explained and described as it was used in this study.

### Tuning of Hyperparameter (Nested CV Inner Loop)

Two major hyperparameters were considered to be tuned in this study and this included the number of variables tried at each node denoted as *mtry* in the RF package (23) and the maximum number of unique observation required to split a node (*minsplit* or *nodesize* in the RF package). In RF, *mtry* is considered a major and central hyperparameter to be tuned; therefore, the *mtry* was tuned in this study using the R syntax in the package ‘*randomForestSRC*’ (23). Next, the number of unique observations was used to determine the amount of observation to be drawn for each training tree; this process was also performed using the R syntax in the package ‘*randomForestSRC*.’ The optimal tuning of hyperparameters could then be determined using OOB prediction, the lower the estimated OOB prediction the better the selected tuning of hyperparameters.

### Predictive Accuracy Measure (Nested CV Outer Loop)

The accuracy indices of the TRSF and other existing techniques are presented in this section. The predicted risk survival outcomes were assessed by cross-validation of the IBS (28). At an individual time *t*, the Brier score (BS) is the square of the difference in the observed survival status (i.e. 1 if uncensored at time *t* and 0 if censored at time *t*) and the based model prediction of survival probability at time *t*. The estimation prediction accuracy measurement of the BS is given as follows; suppose *M* is the number of observations in the testing dataset, for time *t* > 0 then the inverse probability of censoring weighted BS is given in equation (10):


(Equation 10) $$\hat{BS}(t)=\frac{1}{M}\sum_{i=1}^{M}\left\{\begin{array}{c} {\left(\hat{S}(t,x_{i})\right)}^{2}.\text{I}(y_{i}<t,\delta_{i}=1).{\left(\hat{G}(y_{i}|x_{i})\right)}^{-1}+{\left[1-\hat{S}(t,x_{i})\right]}^{2}.\\ \text{I}(y_{i}>t,\delta_{i}=0).{\left(\hat{G}(y_{i}|x_{i})\right)}^{-1} \end{array}\right\},$$

for all individuals *i* in the testing dataset. Where *Ŝ*(*t*,*x_i_*) is the predicted probability of survival of an individual *i* at time *t*, *x_i_* is the covariate of individual *i, y_i_* is the number of an individual at risk and *Ĝ*(*y_i_*|*x_i_*) is the estimated probability of censoring.

Additionally, the BS estimate $\left(\hat{BS}(t)\right)$ is time-dependent; therefore, the integration of the baseline to the maximum observed event time is necessary for the purpose of direct comparison. Therefore, the defined IBS estimate is provided in equation (11), as follows:


(Equation 11) $$\hat{BS}(T)=\frac{1}{T}\int_{0}^{T}\hat{BS}(t)dt.$$

where *T* is the maximum observed event time.

## Numerical Results and Discussion

In this section, we present an explanatory example that highlights the similarities and differences in TRSF analysis and classical methods. The real-life data used were from the Mayo Clinic trial in primary biliary cirrhosis (PBC) of liver transplants conducted from 1974 to 1984. A total of 424 PBC patients referred to the Mayo Clinic during that 10-year period met eligibility criteria for the randomised placebo-controlled trial of the drug D-penicillamine. The first 312 cases in the dataset participated in the randomised trial and contained largely complete data. The additional 112 cases did not participate in the clinical trial but consented to have basic measurements recorded and to be followed for survival. Six of those cases were lost to follow-up shortly after diagnosis, so the data here are from an additional 106 cases as well as the 312 randomised participants. A careful data cleaning was done on the data to remove some noise data, and the row with the most missing observations and these processes reduced the data to 312 observations (23). The description of this dataset is provided in Table 1 for a better understanding of this real-life data.

In this dataset, there were 16 covariates: ten numerical-data, two factor-data and four binary-data. All these covariates were used in fitting the parametric, semi-parametric and non-parametric TRSF models. The results of the parametric models were compared with the Cox-PH model using AIC criteria as shown in Table 2.

The results of the AIC shown in Table 2 revealed that the Cox-PH model was better than the three fitted parametric models since its AIC was smaller compared with the others. Moreover, as pointed out, there were 16 covariates and all of them have been proven to influence the survival of a patient with liver transplant by medical practitioners. However, in all the parametric fitted models, the maximum covariates that influenced the survival of patients from this disease was five, while for the Cox-PH model, it was three covariates and by the virtue of the principle of parsimony, Cox-PH still seemed to be the best. Sometimes, the interest of clinical researchers may not only be the influential covariates but also how these covariates are important to the survival of patients from the liver transplant. In this regard, the proposed RSF can account for the effect, association and importance of all these covariates on the survival of patients. The results of the tuning hyperparameters are presented in Table 3. The purpose of obtaining these results was for selecting the best tuning parameters. The results (Table 3) revealed that the best number of variables needed for each node was four and the unique observation needed for splitting a node was 15. These optimal results were then used to train our final model and the prediction accuracy (IBS) of the final model was then compared with other existing methods.

**Figure 1:**
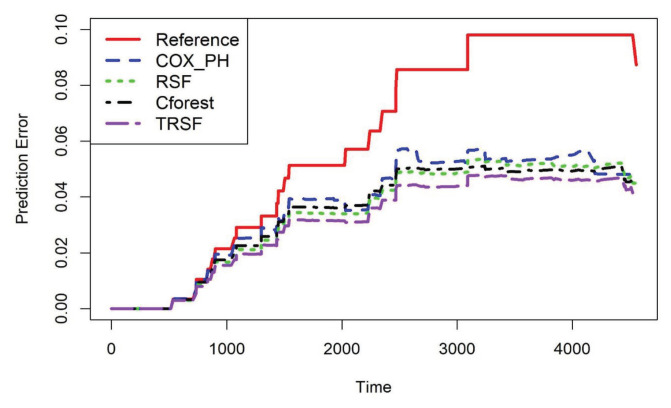
Plot of prediction error rate against survival time

Next, Table 4 presents the results of IBS for the TRSF, Cox-PH model, Cforest, RSF and the reference model (29). Here, the IBS is used to measure the prediction accuracy of these models for comparison purposes and the smaller the IBS the better the model. The results revealed a slight improvement in the predictive accuracy of the TRSF model over all the highlighted existing methods. The latter was also plotted against time as shown in Figure 1.

In Figure 1, the reference model (Kaplan-Meier) is represented by the solid line (red), the dashed line (blue) stands for the Cox-PH model, the RSF is represented by the dotted line (green), the dot-dash line (black) stands for Cforest and the TRSF is represented by a long-dash line (purple). It can be deduced from this figure that the TRSF has the lowest prediction error rate and therefore can be considered the best model of the five.

**Figure 2:**
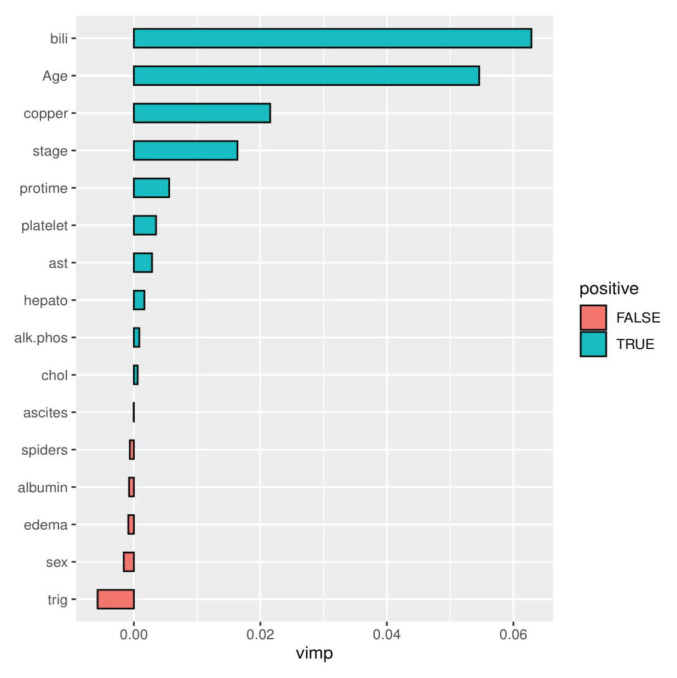
Plot of VIMP

Further analysis was done on the TRSF model via the variable selection technique. The variable selection was done using variable importance (VIMP) and minimal depth. A property derived from the dependence and partial dependence plots to aid the interpretation of RSF methods for both prediction and information retrieved specifically in time to event datasets. We fit RSF on the covariates and then checked whether we had covariates that contributed to the model positively or negatively. The results that revealed the importance and association of the covariates to the survival of patients with liver transplants are presented in increasing order in Table 4. The results of the variable importance are further presented graphically in Figure 2.

We can see from Table 5 and Figure 2 that some covariates contributed positively to the survival of patients who received a liver transplant, while some contributed negatively. The covariates that contributed positively are bilirubin, age, copper, protime, platelet, aspartate aminotransferase, stage, alkaline phosphatase and triglycerides, respectively, while those that contributed negatively are ascites, serum cholesterol, sex, spiders, edema, hepatomegaly and serum albumin, respectively.

**Figure 3:**
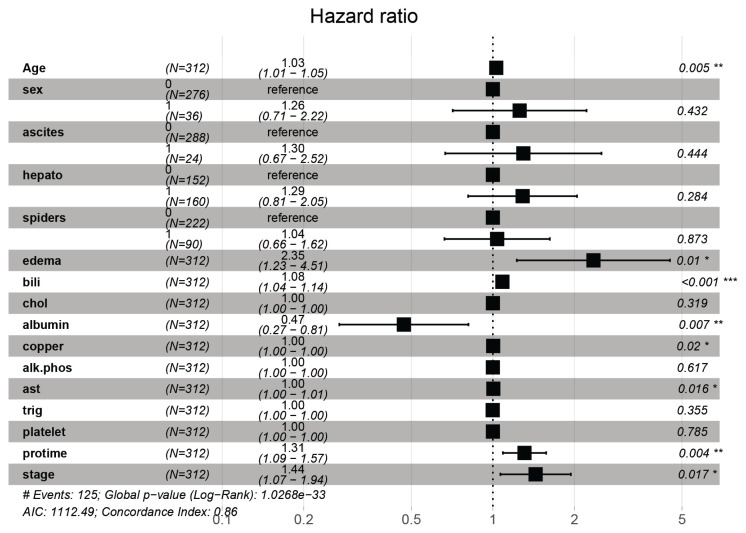
A Forest plot showing HR, 95% CI and *P*-values associated with variables considered in the analyses with time to the PBC of the liver transplant as the dependent variable

In order to shed more light on the effect of the covariates associated with time to the PBC, hazard ratio (HR), 95% confidence interval (CI) and *P*-values of these variables are presented in Figure 3. The box in the figure represents HR, the horizontal bars that extend from the lower to the upper limits are the 95% CI of the HR estimates and the last column represents the *P*-values of the various covariates, respectively. The results revealed that there was a decrease in the risk of having PBC with a lower albumin level (HR = 0.47; 95% CI = 0.27, 0.81) and significantly associated with the time to PBC (*P*-value = 0.007 < 0.05). Other covariates such as sex, ascites, hepatic, spider, oedema, bilirubin, protime and stages have increasing HRs of time to PBC, while copper, alkaline phosphatase, ast trig and platelets have constant HR over time of PBC.

## Conclusion

In this work, we fitted three different parametric survival analyses to the liver transplant dataset and found that age, sex, platelets, ascites and stage influence patient survival after a liver transplant. Furthermore, the Cox-PH model was also applied to the data and we found comparable results to that of the parametric model. We used AIC to identify the best model among the classical fitted models; interestingly, the Cox-PH model was identified as the best model.

The results of this study indicate that it is fitting to compare the RF methodologies (RSF and Cforest) with tuned hyperparameters (TRSF). The results of the comparative study revealed that the tuned hyperparameters (TRSF) produced more accurate predictions compared with other existing RF methodologies (RSF and Cforest), including the classical survival methodologies considered based on the examined dataset.

In addition, we introduced an RSF model to this dataset to account for the importance and association that exist between the covariates and patient survival. The results of TRSF revealed that some covariates are associated with the survival of patients with liver transplants and that these covariates include sex, oedema, spiders, albumin and triglycerides, while others do not influence the survival of patients with liver disease. Finally, we compared RSF with the Cox-PH model using IBS and found that TRSF performs better than any other classical model.

## Figures and Tables

**Table 1:** Description of the liver transplant data

Variable name	Description	Type of data
Years	Survival time (years )	Numerical
Status	Event (*F* = censored, *T* = death)	Binary
Treatment	Treatment (DPCA, Placebo)	Factor
Age	Age (years old)	Numerical
Sex	Female = F and Male = M	Binary
Ascites	Presence of ascites	Binary
Hepatom	Presence of hepatomegaly	Binary
Spiders	Presence of spiders	Binary
Oedema	Oedema (0, 0.5, 1)	Factor
Bili	Serum bilirubin (mg/dL)	Numerical
Chol	Serum cholesterol (mg/dL)	Numerical
Albumin	Albumin (gm/dL)	Numerical
Copper	Urine copper (ug/day)	Numerical
Alk	Alkaline phosphatase (U/L)	Numerical
Sgot	SGOT (U/mL)	Numerical
Trig	Triglycerides (mg/dL)	Numerical
Platelet	Platelet per cubic (mL/1000)	Numerical
Protime	Prothrombin time (sec)	Numerical
Stage	Histologic stage	Factor

**Table 2:** Comparison between the fitted parametric models and Cox-PH model

Methods	Degree of freedom	AIC
Exponential	17	427.7262
Weibull	18	417.2082
Lognormal	18	415.6323
Cox-PH	16	185.7233

**Table 3:** Selected optimal tuning hyparameters

*nodesize*	*mtry*	*error*
.	.	.
.	.	.
.	.	.
10	13	0.2648
10	16	0.2745
15	3	0.2552
**15**	**4**	**0.2396**
15	5	0.2927
15	6	0.2830
15	7	0.2653
15	8	0.2999
15	9	0.2830
15	11	0.2731
15	13	0.2940
15	16	0.2635
20	5	0.2943
20	6	0.2635
.	.	.
.	.	.
.	.	.

**Table 4:** IBS value aggregated over 2,000 boostrap cross-validation for the four existing models and TRSF

Model	IBS
Reference (Kaplan and Meier)	0.059
Cox-PH model	0.046
TRSF	**0.044**
Cforest	0.046
RSF	0.045

**Table 5:** Variable important of each covariate

Covariate	Variable importance
Age	0.0248
Bilirunbin	0.0240
Copper	0.0098
Aspartate aminotransferase	0.0057
Platelet	0.0047
Protime	0.0038
Stage	0.0036
Hepatomegaly	0.0033
Serum cholesterol	0.0018
Alkaline phosphatase	0.0005
Ascites	0.0001
Sex	−0.0002
Oedema	−0.0003
Spiders	−0.0008
Serum albumin	−0.0020
Triglycerides	−0.0022
